# Linking Academic Procrastination, Test Anxiety, and Academic Achievement Among University Students

**DOI:** 10.1192/j.eurpsy.2026.11310

**Published:** 2026-07-31

**Authors:** I. Kececi, C. Tarim, G. Ozturk, B. Toy, A. Topuzoglu

**Affiliations:** 1 Marmara University Medical School; 2 Uskudar University Medical School, Istanbul, Türkiye

## Abstract

**Introduction:**

Academic procrastination is a common behavior among university students and can negatively affect academic performance. At university, this behavior can be seen as delaying studying for classes, leaving homework to the last minute, missing deadlines for essential projects, and not preparing for exams until the last minute. Anxiety theory suggests that anxiety works as a mental response to avoid future catastrophic experiences related to a particular situation and functions as a cognitive avoidance maneuver in response to perceived threats. Encountering anxiety-provoking situations can cause intense anxiety and fear in the individual. To avoid these feelings and related symptoms, they may use avoidance behaviors.

**Objectives:**

This study aims to examine the relationship between test anxiety and academic procrastination among students.

**Methods:**

Students aged 18 or older who provided informed consent, could read and understand Turkish, and were currently studying at Marmara University were included in the study. The Westside Examination Anxiety Scale (WSKÖ), consisting of 11 items on a five-point Likert scale, and the Academic Procrastination Scale (AEÖ), consisting of 19 items (12 negative and 7 positive) were used. Survey data were analyzed statistically, and relationships between variables were evaluated. Repeated-measures ANOVA and the Student-Newman-Keuls test were used for within-group comparisons. One-way ANOVA and Tukey’s multiple comparison test were used for between-group comparisons. A p-value of <0.05 was accepted as statistically significant.

**Results:**

The mean age of the 421 participants was 21.8 (±2.067). Of the participants, 42% were women and 57% were men. Only 8.1% had no siblings. A total of 43.5% lived in a metropolitan city until the age of 12. While 17.3% lived alone, most lived with family or friends. Also, 89.1% had never repeated a grade. There was no significant relationship between anxiety and procrastination scores and variables such as gender, parental education level, number of siblings, living place, who they lived with, faculty, grade level, GPA, or grade repetition history (p>0.05). There was also no significant correlation between test anxiety scores and academic procrastination scores (p=0.808).

**Conclusions:**

Exam anxiety and academic procrastination are widely studied topics. While some previous studies report a link between these two, others, including this study, do not. Given how common these issues are and the mixed findings in the literature, more research is needed to understand better the factors involved. With more information, suitable support methods and strategies can be developed for students when needed.

**Disclosure of Interest:**

None Declared

